# Supplementary figures and images for: N-cadherin directs the collective Schwann cell migration required for nerve regeneration through Slit2/3-mediated contact inhibition of locomotion
Source: eLife. 2024 Apr 9;13:e88872. doi: 10.7554/eLife.88872 (PMC11052573; doi:10.7554/eLife.88872)

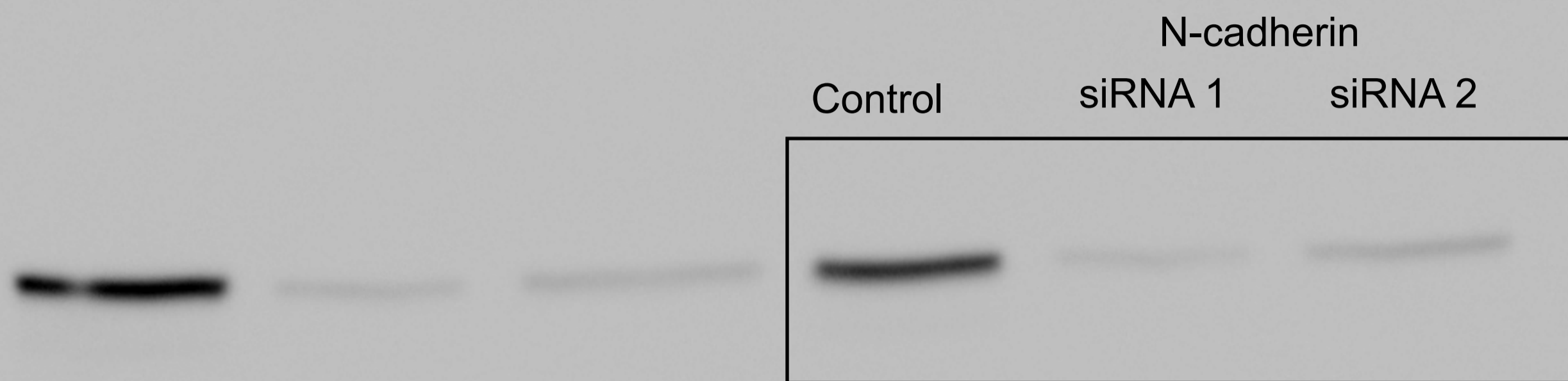

Supplement: Figure 1—figure supplement 1—source data 2. [file elife-88872-fig1-figsupp1-data2.zip › Figure 1-Source Data 2/N-cadherin annotated.pdf]

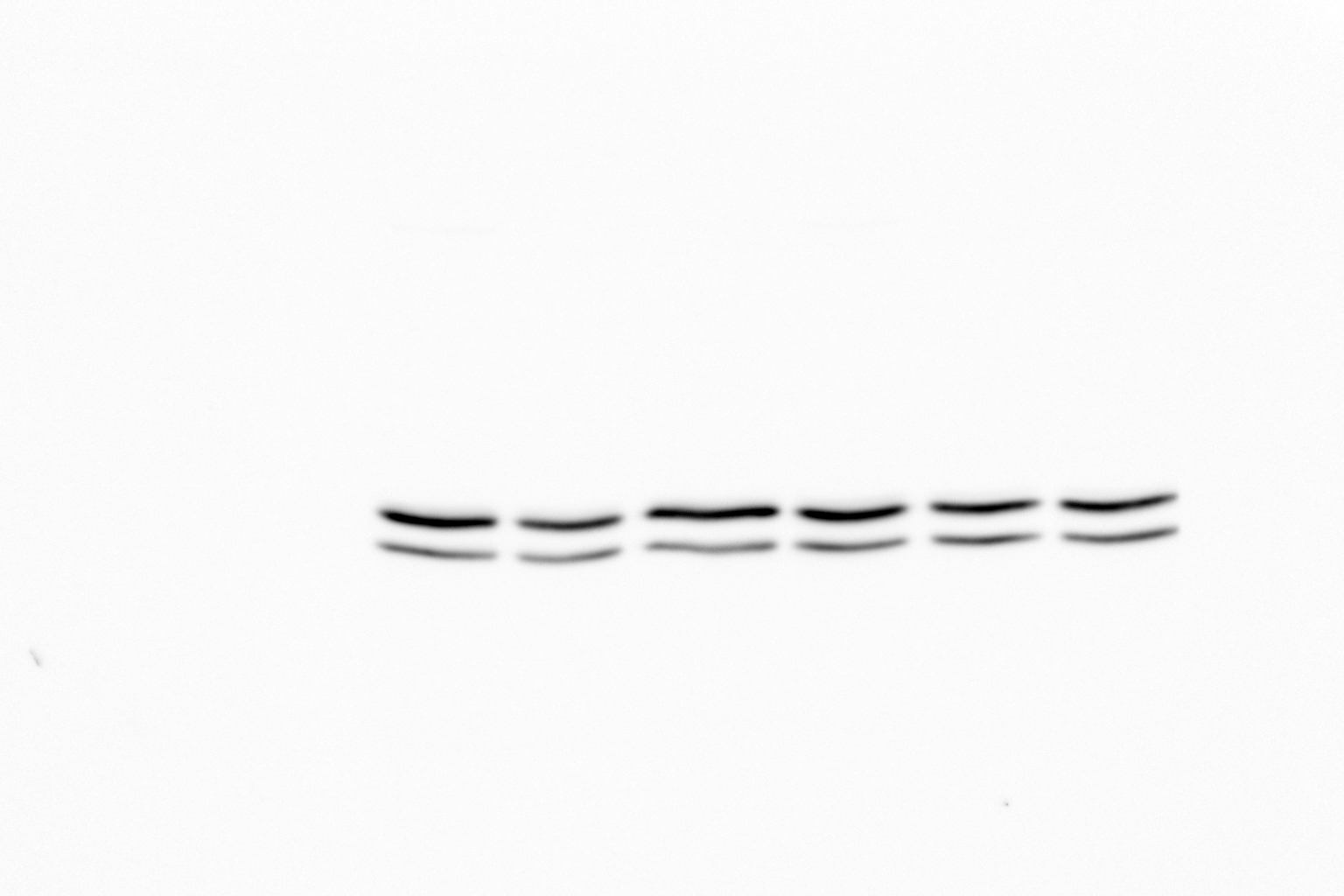

Supplement: Figure 1—figure supplement 1—source data 3. [file elife-88872-fig1-figsupp1-data3.zip › Figure 1-Source Data 3/ERK.tiff]

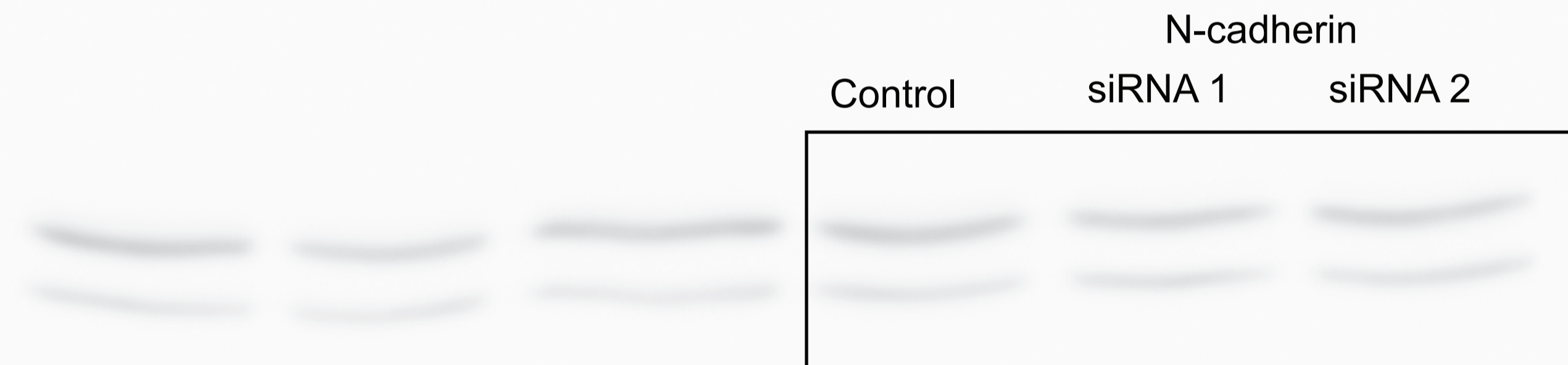

Supplement: Figure 1—figure supplement 1—source data 4. [file elife-88872-fig1-figsupp1-data4.zip › Figure 1-Source Data 4/ERK annotated.pdf]

— — — — — — — — — —

alpha-catenin

Control

siRNA 1

siRNA 2

siRNA 1+2

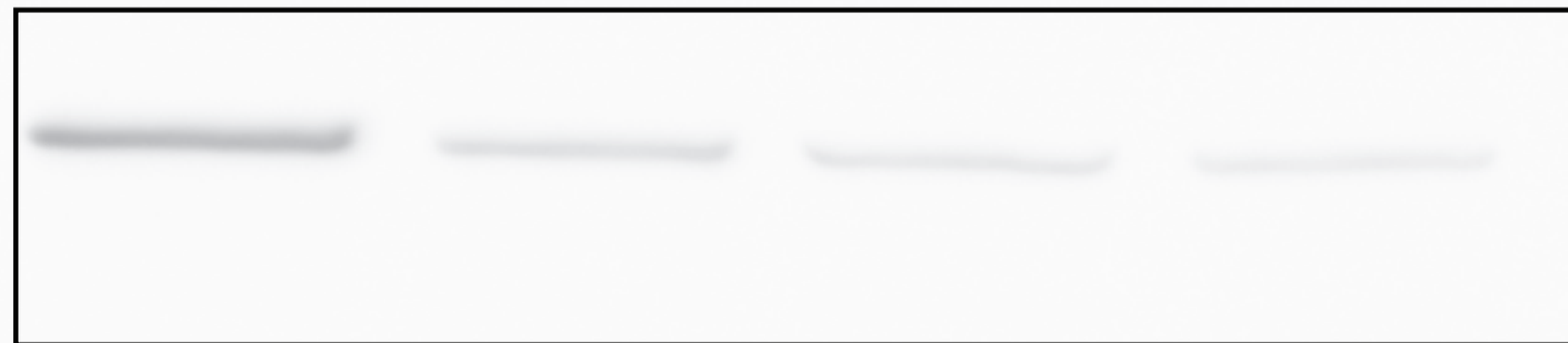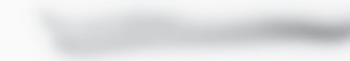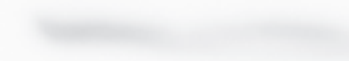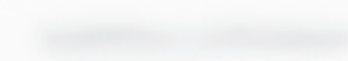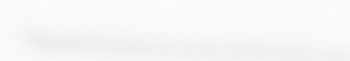

Supplement: Figure 2—figure supplement 1—source data 1. [file elife-88872-fig2-figsupp1-data1.zip › Figure 2-Source Data 1/alpha-catenin S2c.pdf]

alpha-catenin

Control

siRNA 1

siRNA 2

siRNA 1+2

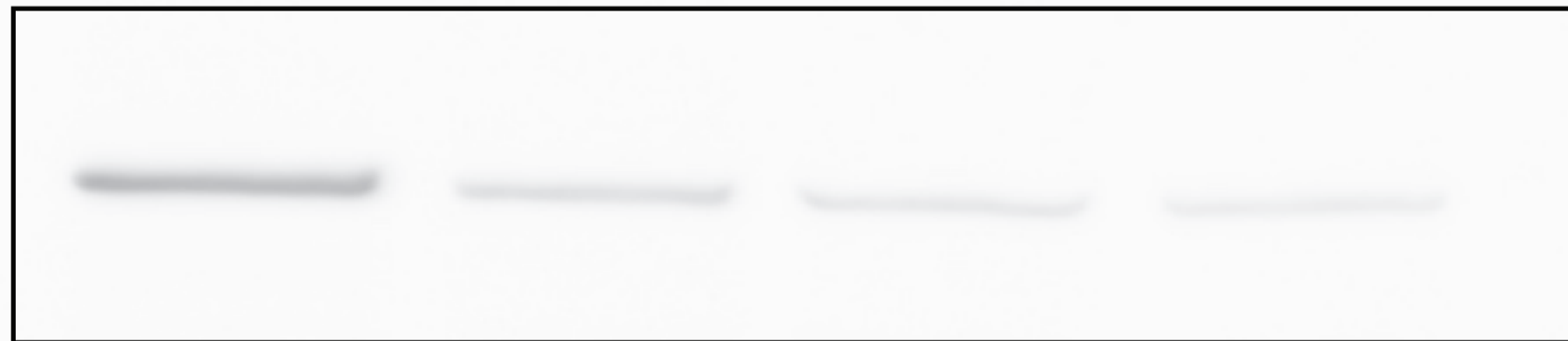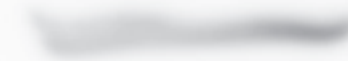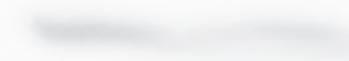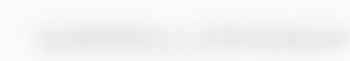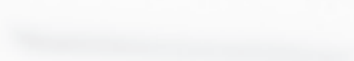

Supplement: Figure 2—figure supplement 1—source data 2. [file elife-88872-fig2-figsupp1-data2.zip › Figure 2-Source Data 2/alpha-catenin S2c annotated.pdf]

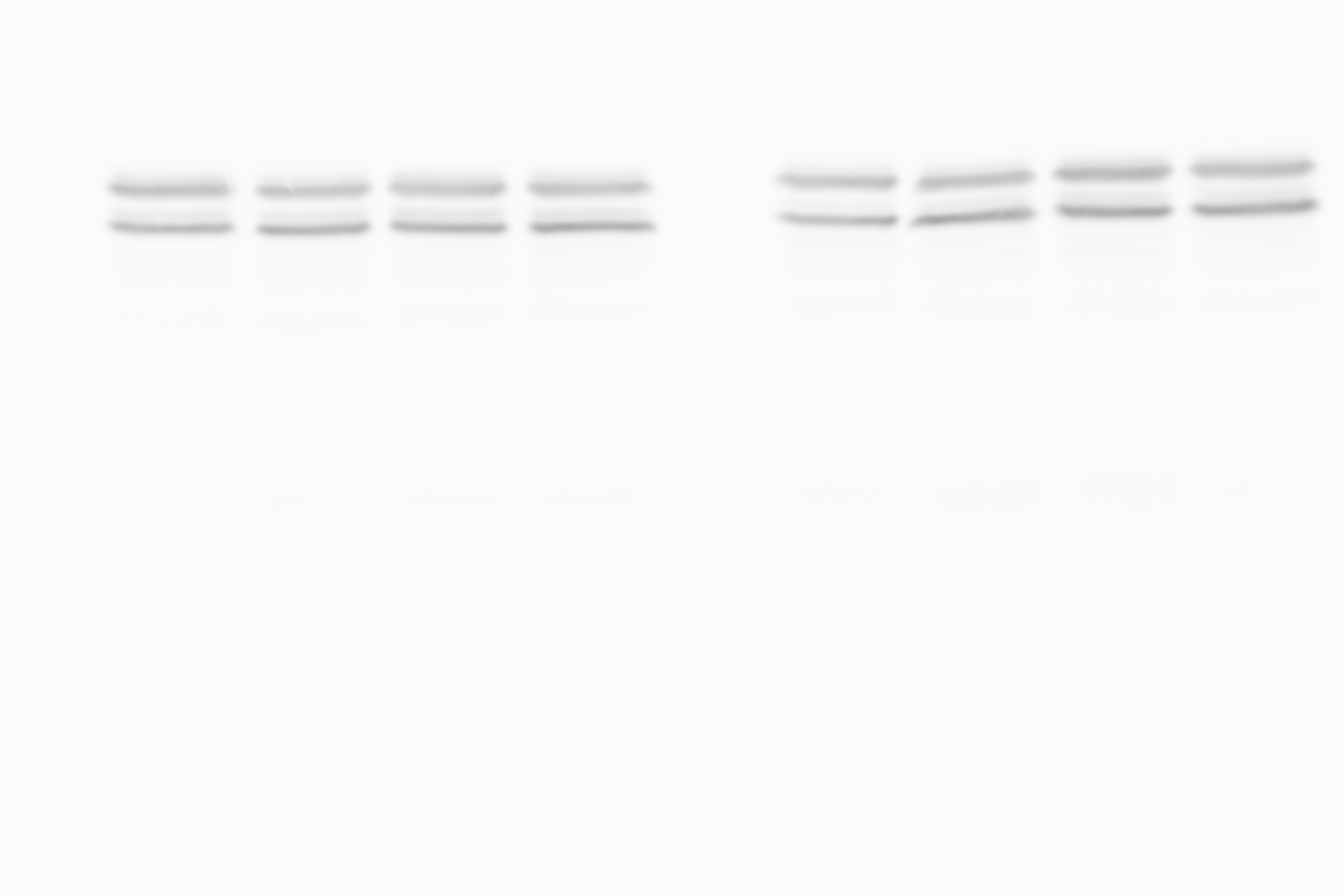

Supplement: Figure 2—figure supplement 1—source data 3. [file elife-88872-fig2-figsupp1-data3.zip › Figure 2-Source Data 3/ERK S2c.pdf]

alpha-catenin

Control

siRNA 1

siRNA 2

siRNA 1+2

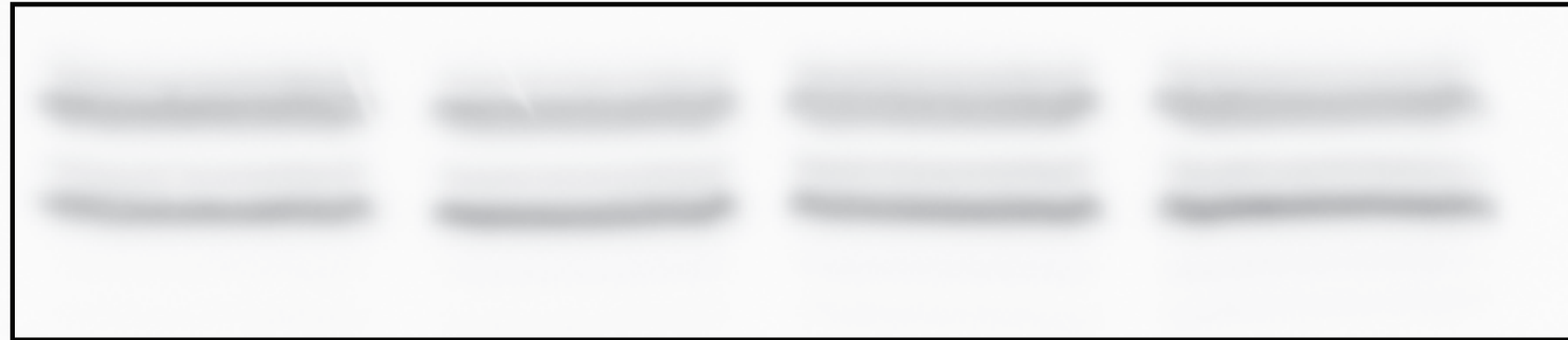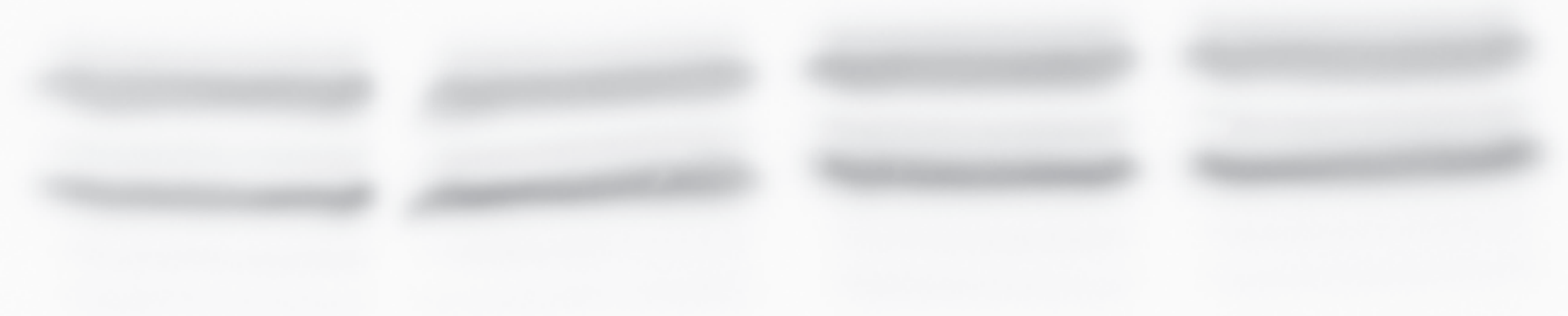

Supplement: Figure 2—figure supplement 1—source data 4. [file elife-88872-fig2-figsupp1-data4.zip › Figure 2-Source Data 4/ERK S2c annotated.pdf]

p120-catenin

Control

siRNA 1

siRNA 2

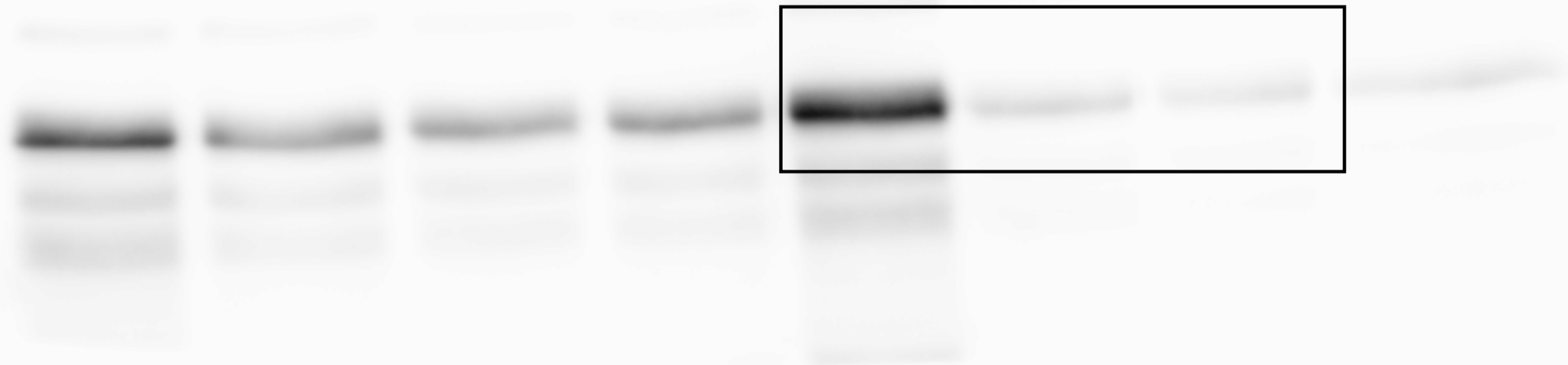

Supplement: Figure 2—figure supplement 1—source data 6. [file elife-88872-fig2-figsupp1-data6.zip › Figure 2-Source Data 6/p120s2e annotated.pdf]

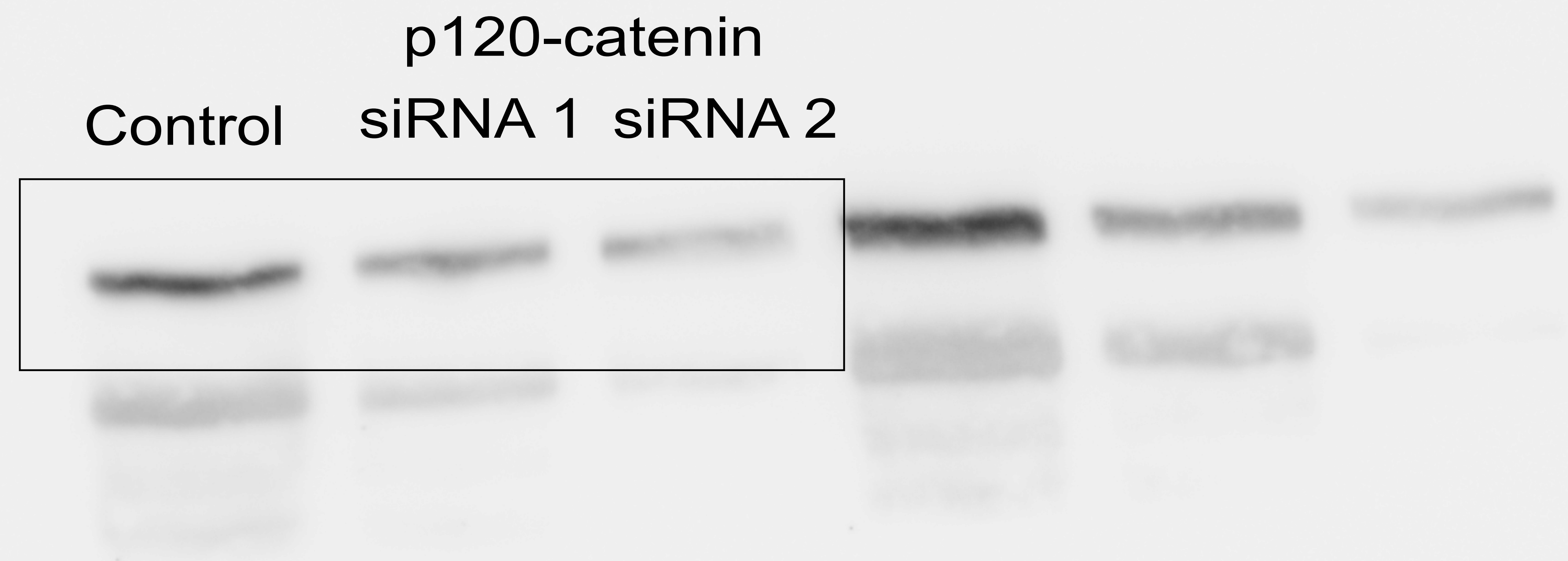

Supplement: Figure 2—figure supplement 1—source data 8. [file elife-88872-fig2-figsupp1-data8.zip › Figure 2-Source Data 8/N-cadherin S2e annotated.pdf]

p120-catenin

| Control | siRNA 1 | siRNA 2 |
|---------|---------|---------|
|---------|---------|---------|

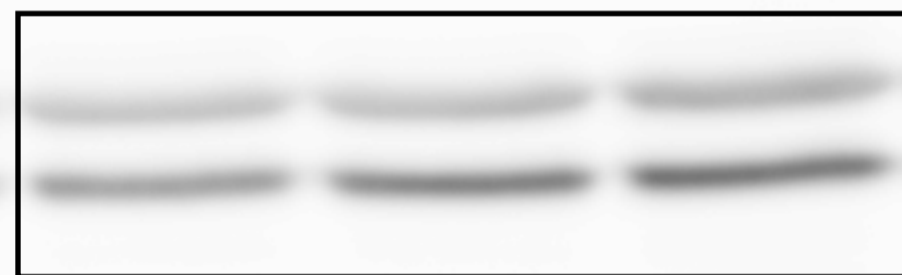

Supplement: Figure 2—figure supplement 1—source data 10. [file elife-88872-fig2-figsupp1-data10.zip › Figure 2-Source Data 10/ERK S2e annotated.pdf]

SiRNA N-CAD

Control      Si1      FL      ICD      ECD

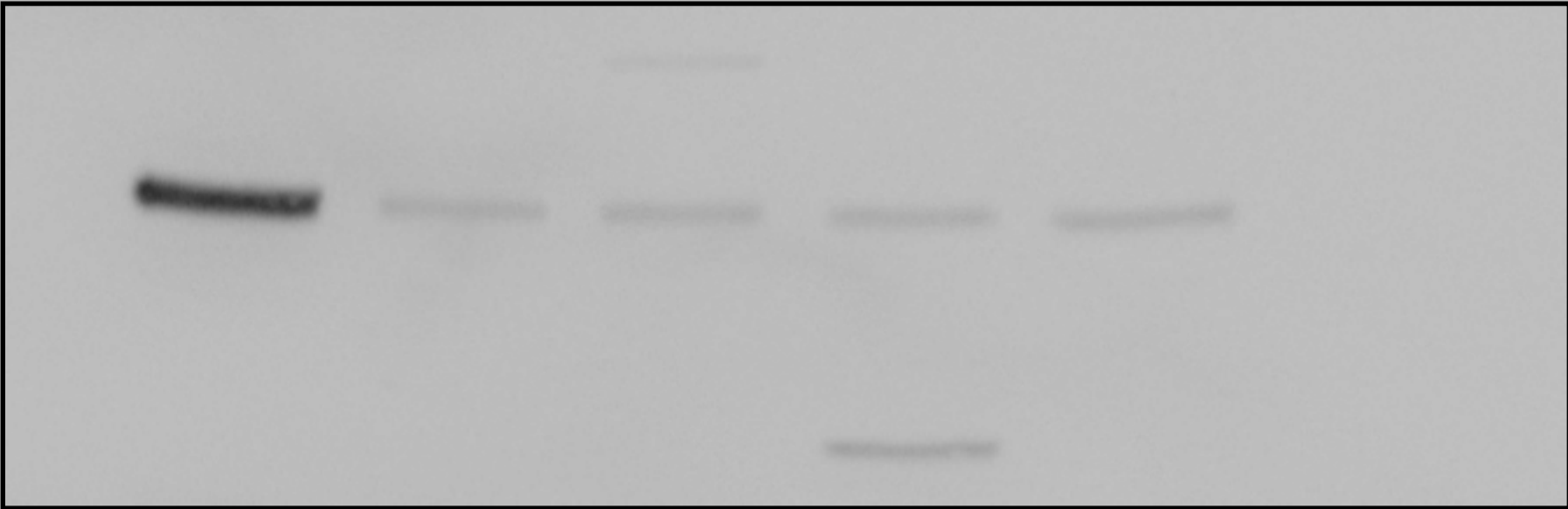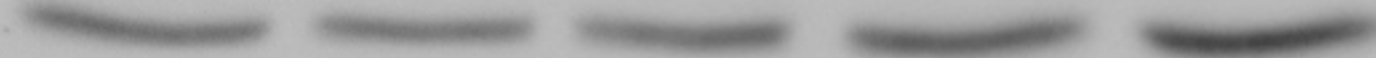

Supplement: Figure 3—source data 2. [file elife-88872-fig3-data2.zip › Figure 3-source data 2/N-cadherin Fig3b annotated.pdf]

SiRNA N-CAD

Control

Si1

FL

ICD

ECD

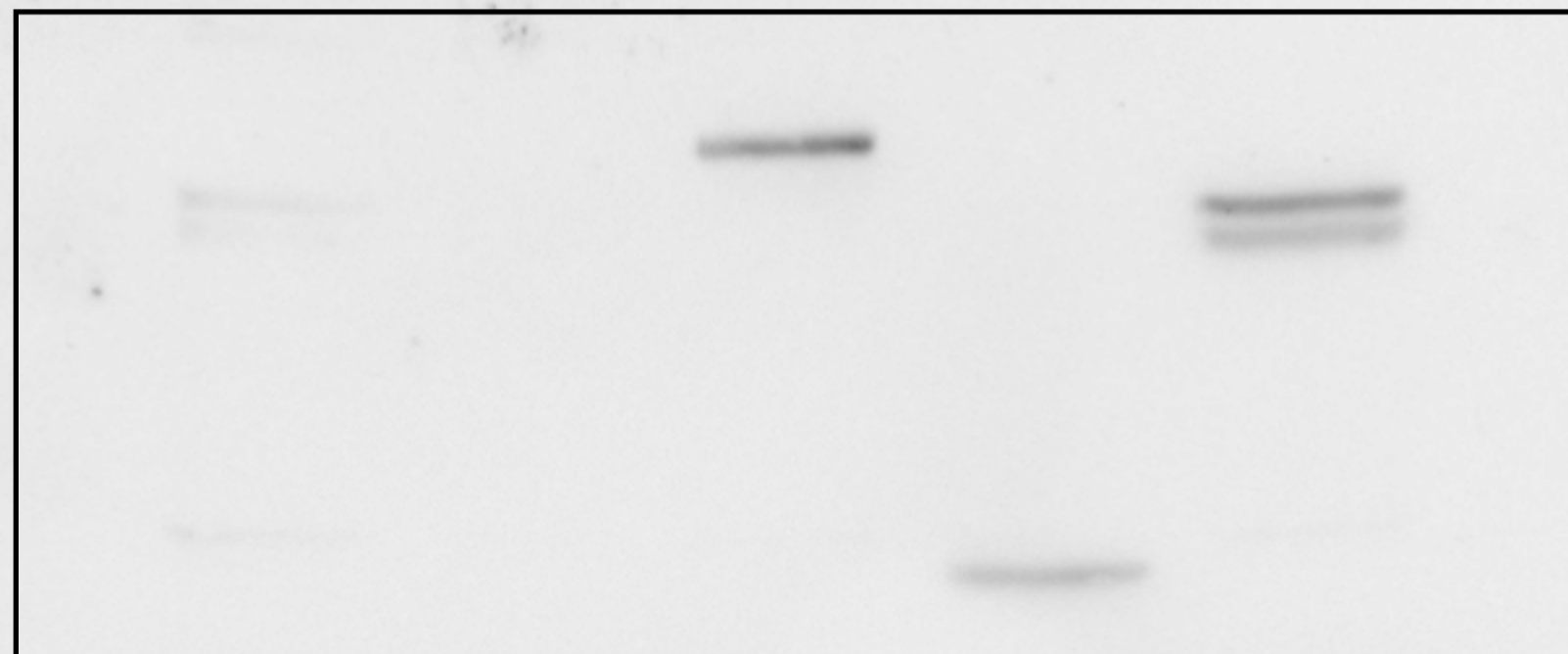

Supplement: Figure 3—source data 4. [file elife-88872-fig3-data4.zip › Figure 3-source data 4/Tomato Fig 3b annotated.pdf]

SiRNA N-CAD

Control

Si1

FL

ICD

ECD

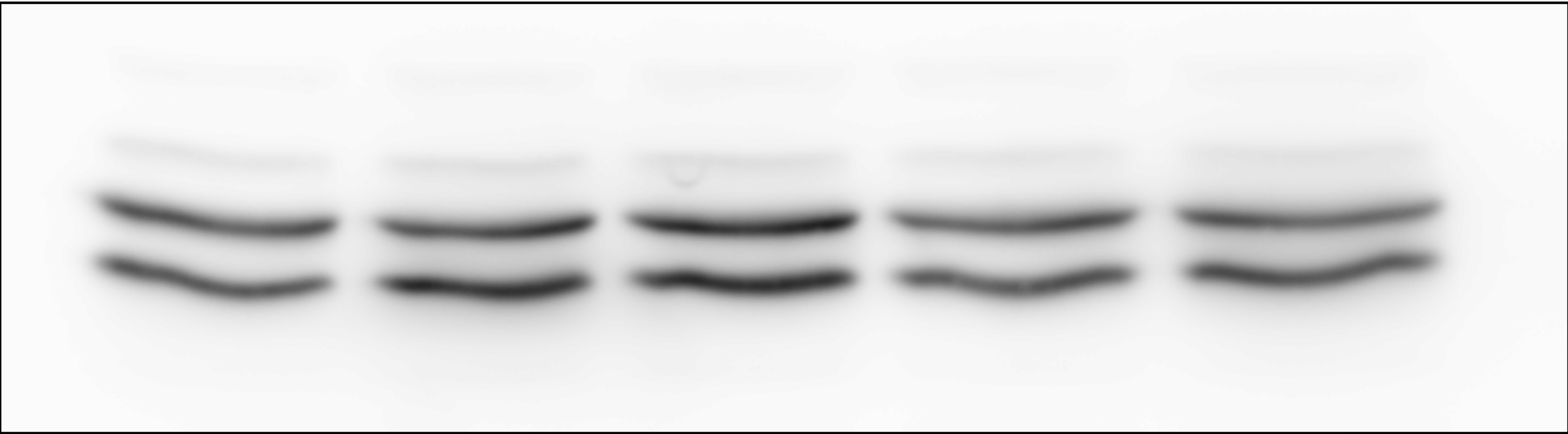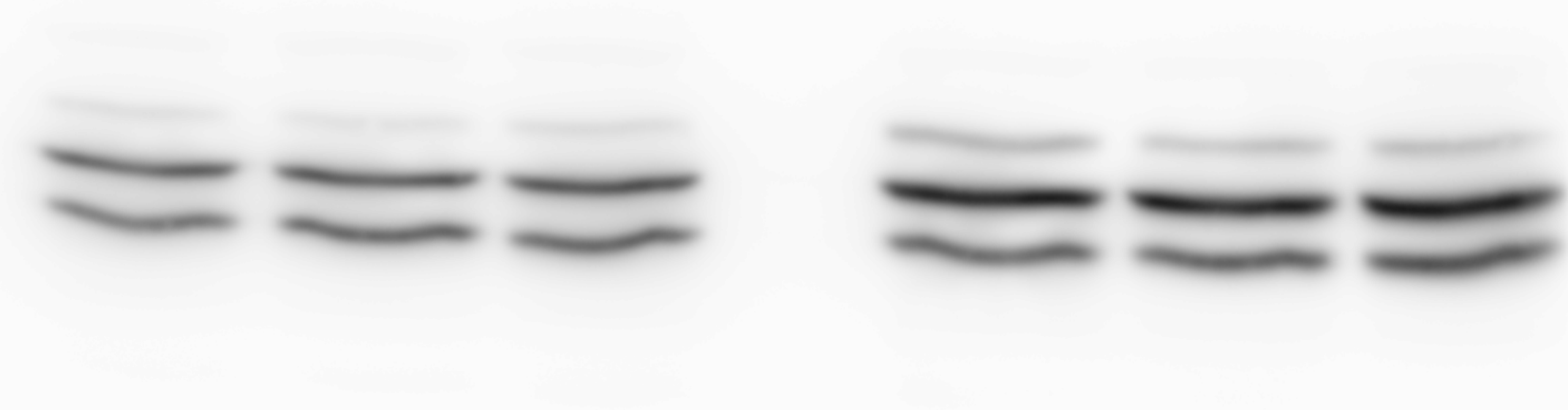

Supplement: Figure 3—source data 6. [file elife-88872-fig3-data6.zip › Figure 3-source data 6/ERK Fig 3b annotated.pdf]

Robo 1

Robo 2

Robo 3

Slit1

Slit2

Slit3

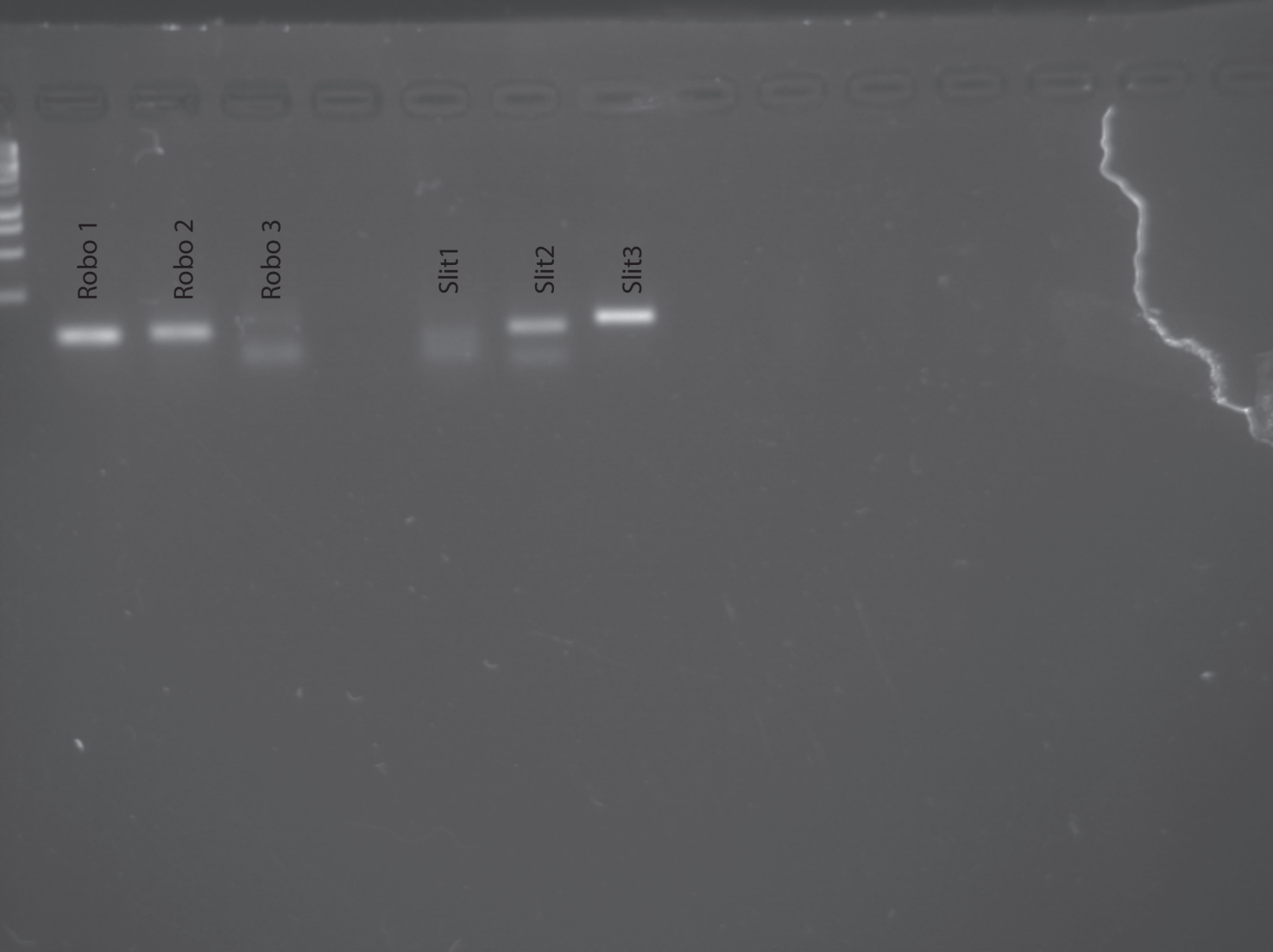

Supplement: Figure 4—figure supplement 1—source data 2. [file elife-88872-fig4-figsupp1-data2.zip › Figure 4-Source data 2/Robo1 2 3 Slit 1 2 3 gel annotated.pdf]

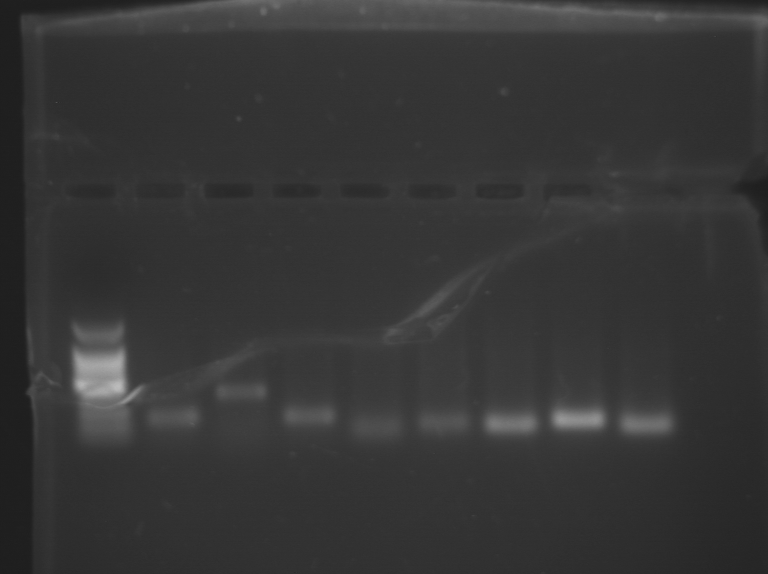

Supplement: Figure 4—figure supplement 1—source data 4. [file elife-88872-fig4-figsupp1-data4.zip › Figure 4-Source data 4/Robo4 gel.TIF]

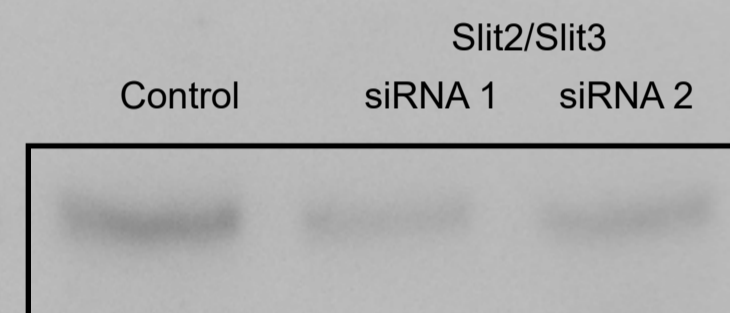

Supplement: Figure 4—figure supplement 1—source data 6. [file elife-88872-fig4-figsupp1-data6.zip › Figure 4-Source Data 6/slit2 S4e annotated.pdf]

Slit2/Slit3

Control

siRNA 1

siRNA 2

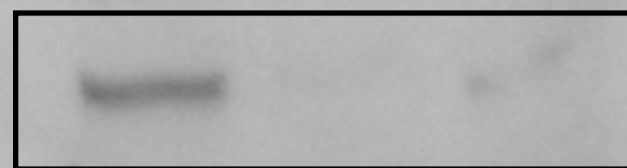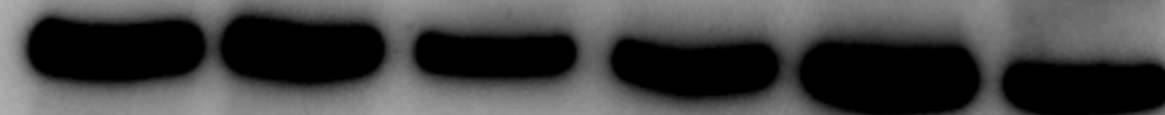

Supplement: Figure 4—figure supplement 1—source data 8. [file elife-88872-fig4-figsupp1-data8.zip › Figure 4-Source Data 8/slit3_up S4e annotated.pdf]

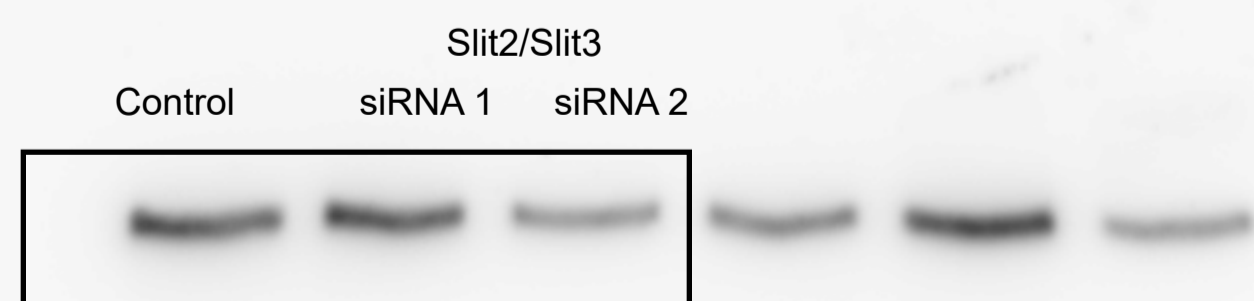

Supplement: Figure 4—figure supplement 1—source data 10. [file elife-88872-fig4-figsupp1-data10.zip › Figure 4-Source Data 10/N-cadherin S4e annotated.pdf]

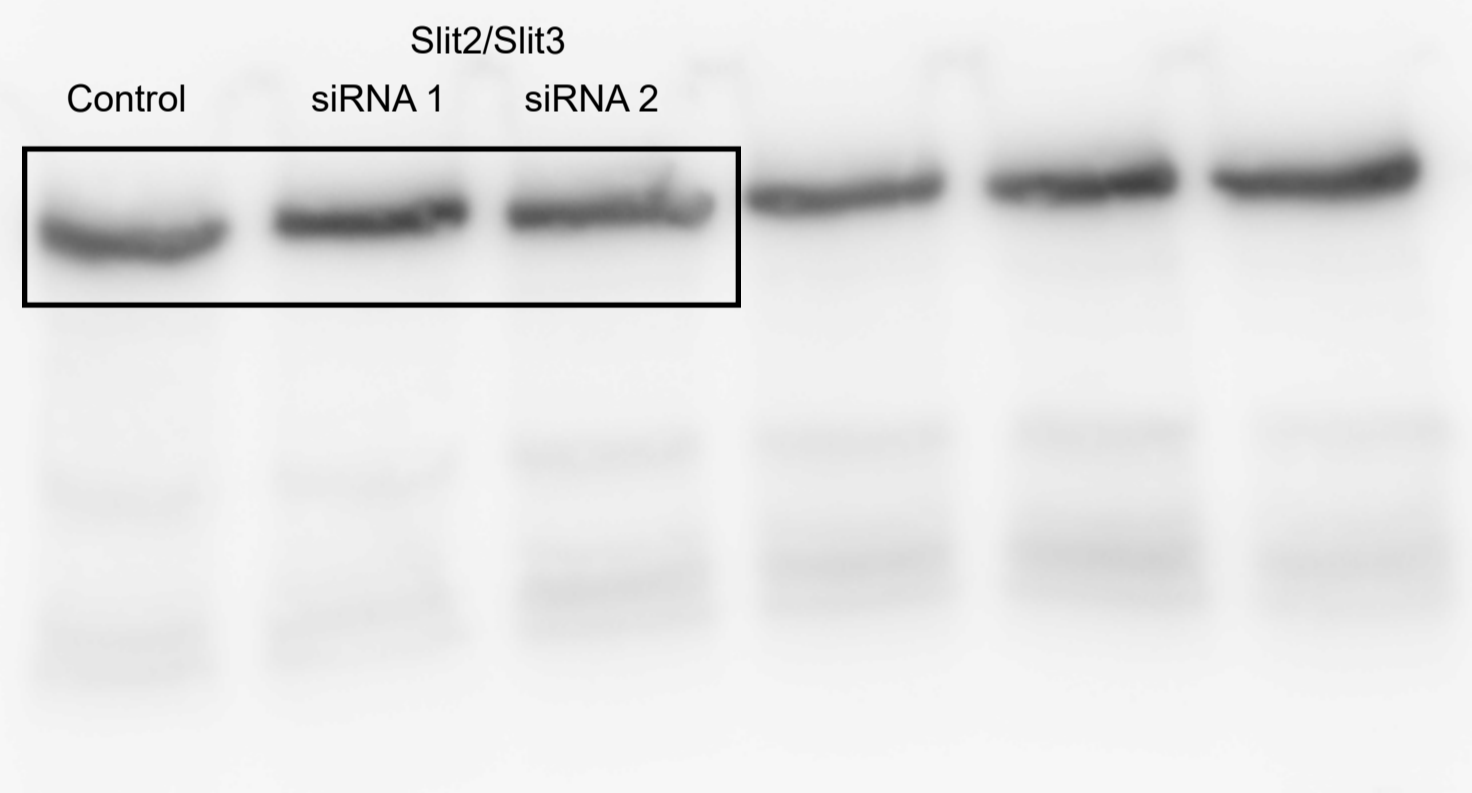

Supplement: Figure 4—figure supplement 1—source data 12. [file elife-88872-fig4-figsupp1-data12.zip › Figure 4-Source Data 12/vinculin_1 S4e annotated.pdf]

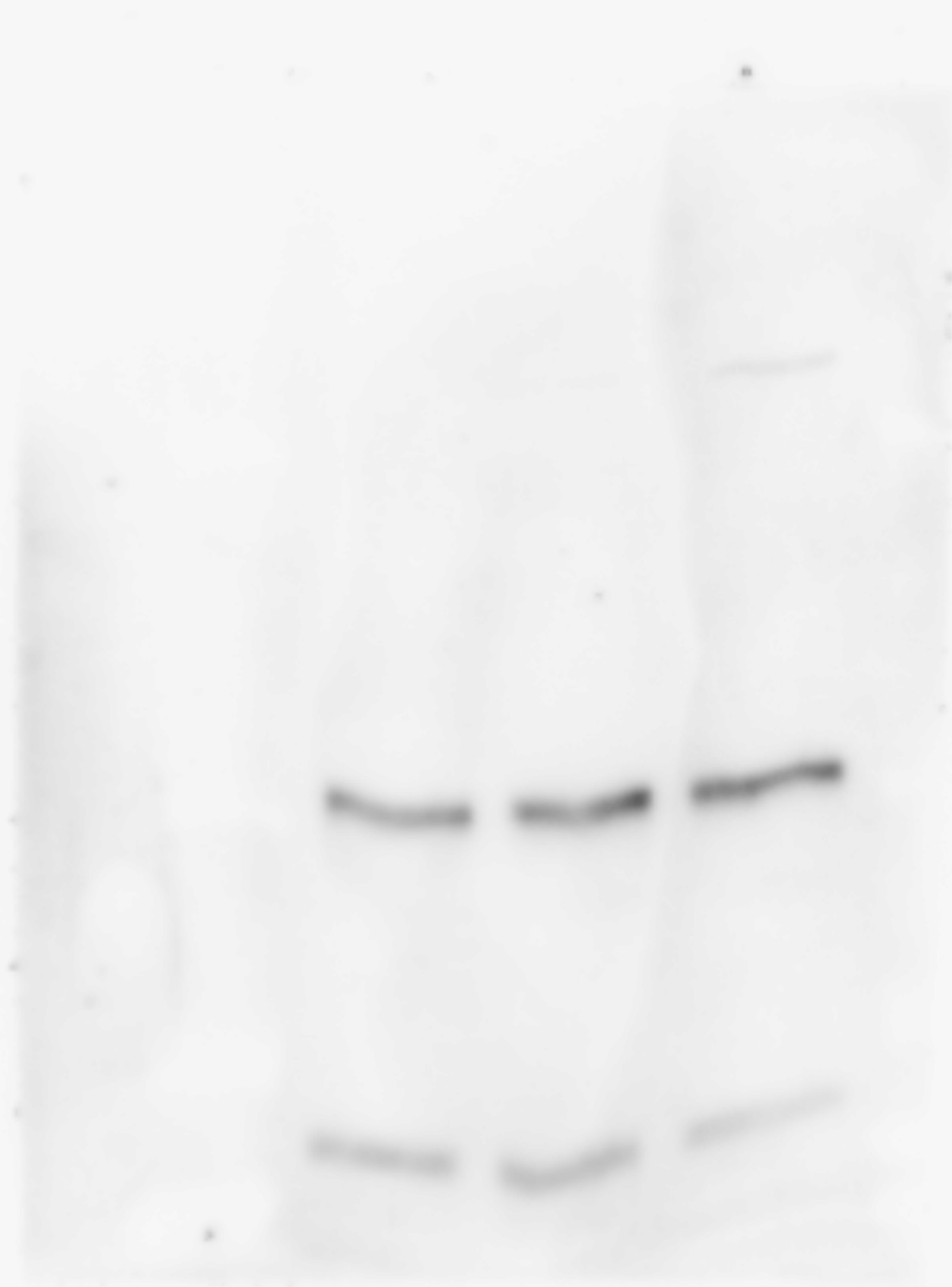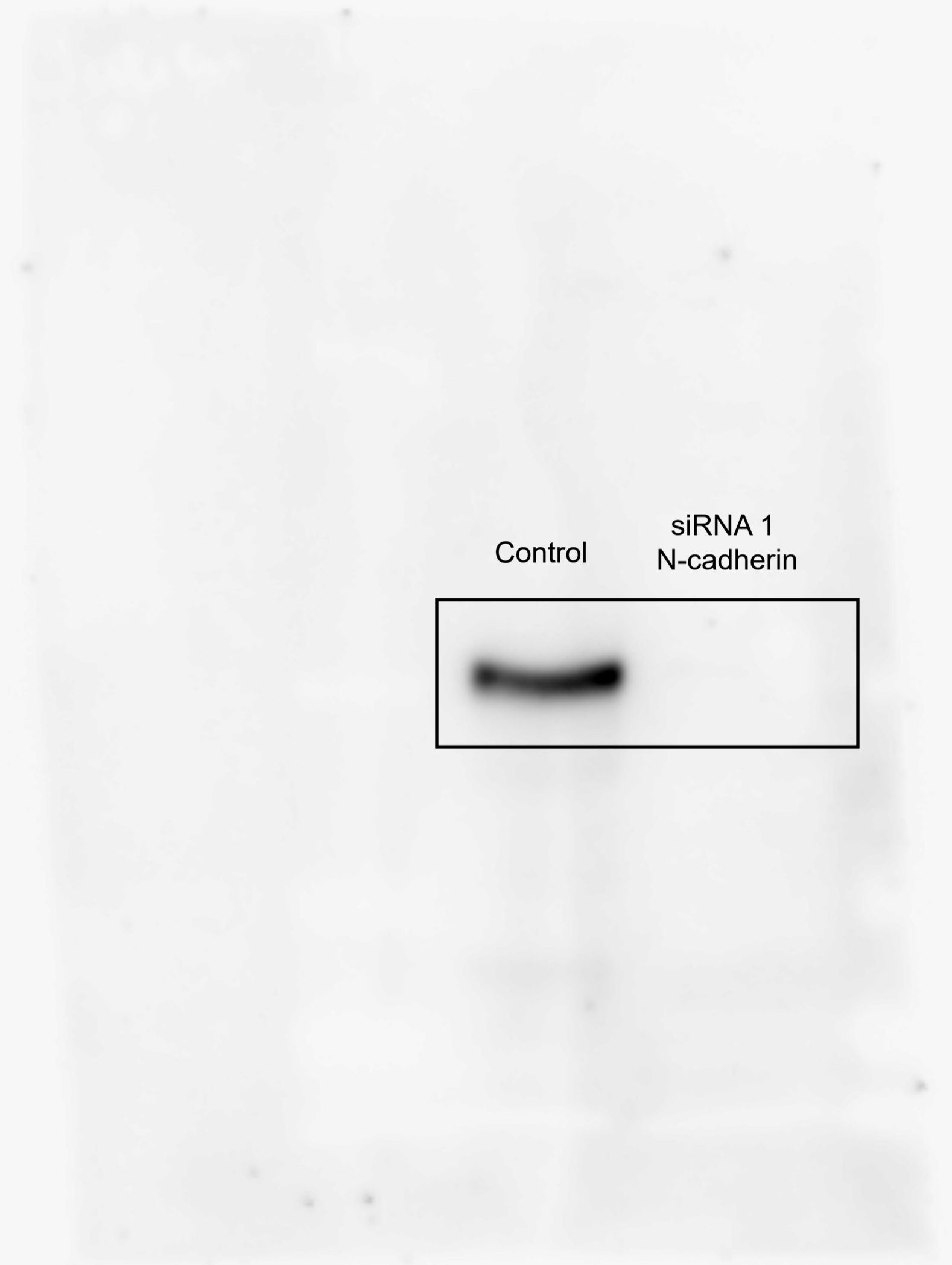

Supplement: Figure 5—figure supplement 1—source data 2. [file elife-88872-fig5-figsupp1-data2.zip › Figure 5-Source Data 2/Ncad_S5a annotated.pdf]

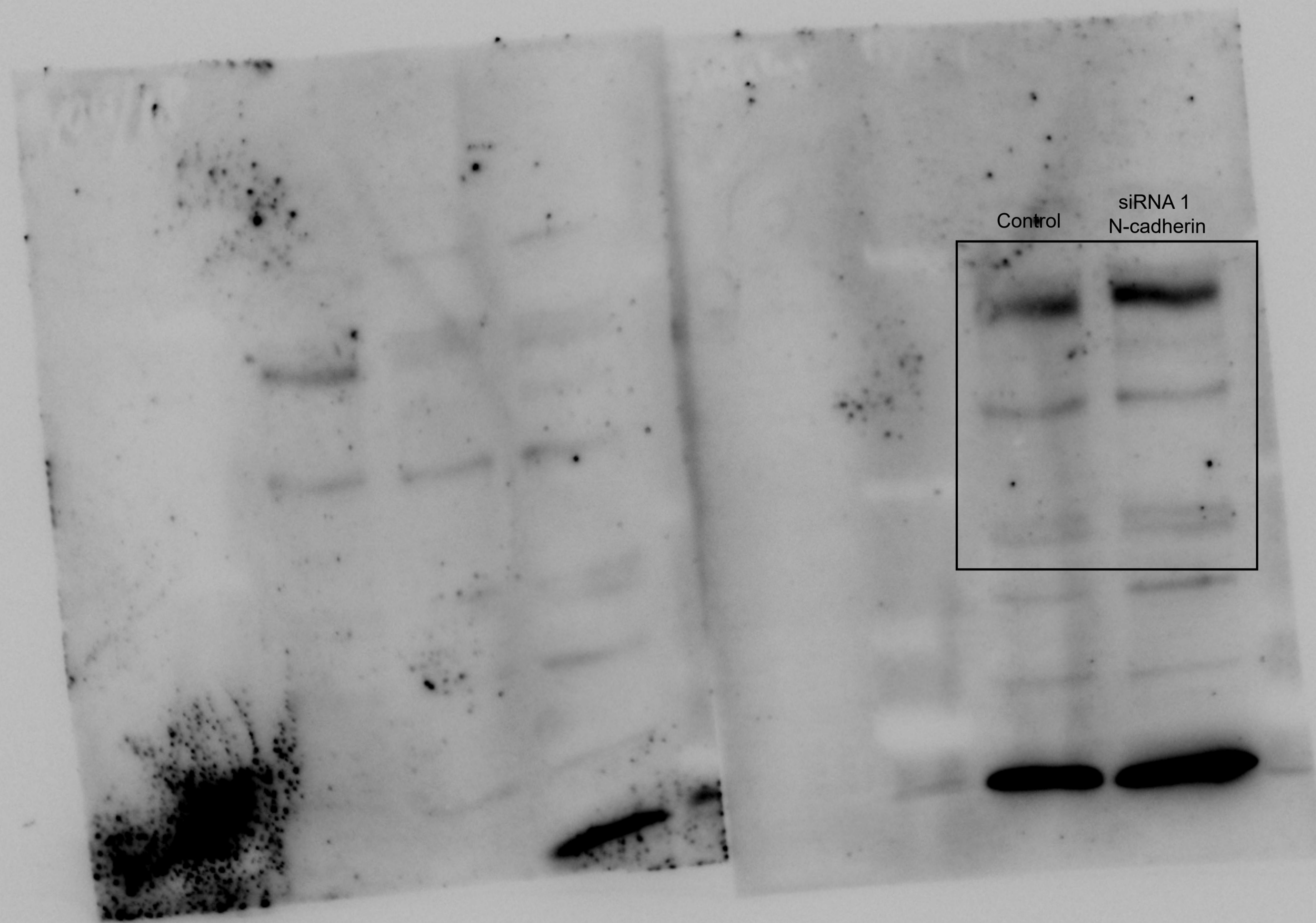

Supplement: Figure 5—figure supplement 1—source data 4. [file elife-88872-fig5-figsupp1-data4.zip › Figure 5-Source Data 4/Slit2 S5a annotated.pdf]

Control

siRNA 1  
N-cadherin

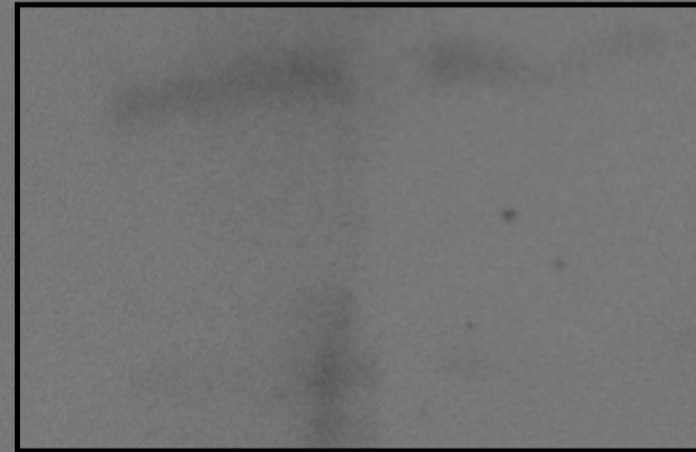

Supplement: Figure 5—figure supplement 1—source data 6. [file elife-88872-fig5-figsupp1-data6.zip › Figure 5-Source Data 6/Slit3 S5a annotated.pdf]

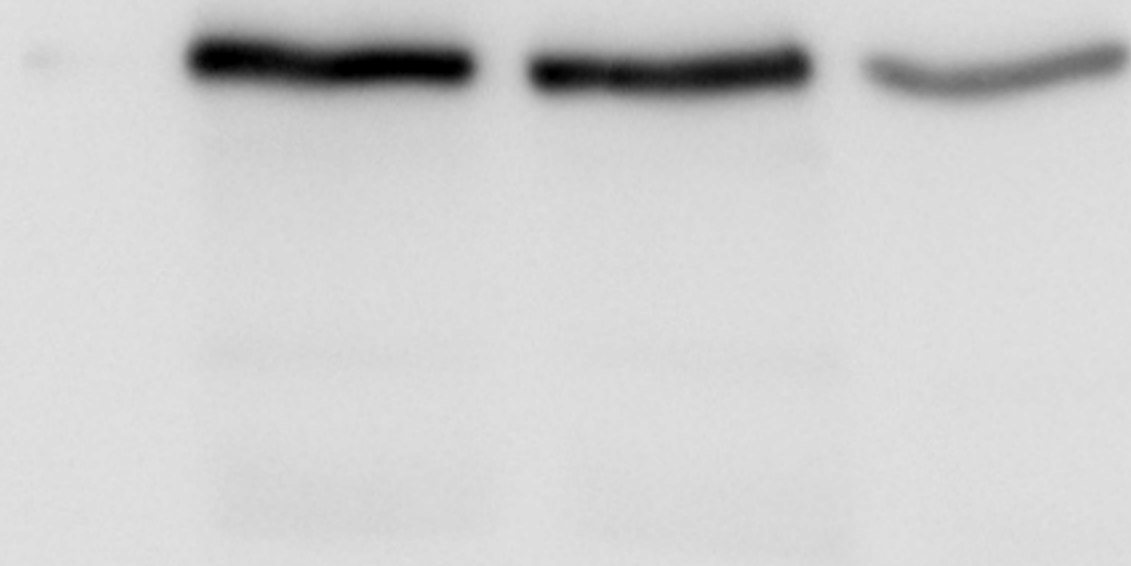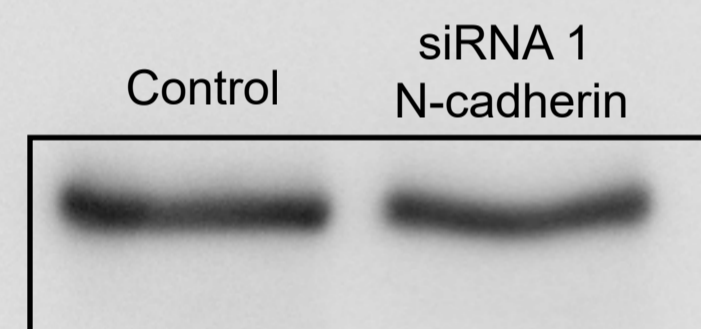

Supplement: Figure 5—figure supplement 1—source data 8. [file elife-88872-fig5-figsupp1-data8.zip › Figure 5-Source Data 8/vinculin S5 annotated.pdf]

Slit2 antibody

| <u>total input</u> |   | IgG | IP:    |  | IgG | IP:    |               |
|--------------------|---|-----|--------|--|-----|--------|---------------|
| +                  | - |     | tomato |  |     | tomato |               |
| +                  | - | +   | +      |  | -   | -      | Tomato        |
| -                  | + | -   | -      |  | +   | +      | N-Cad::Tomato |
| +                  | + | +   | +      |  | +   | +      | Slit-2::Myc   |

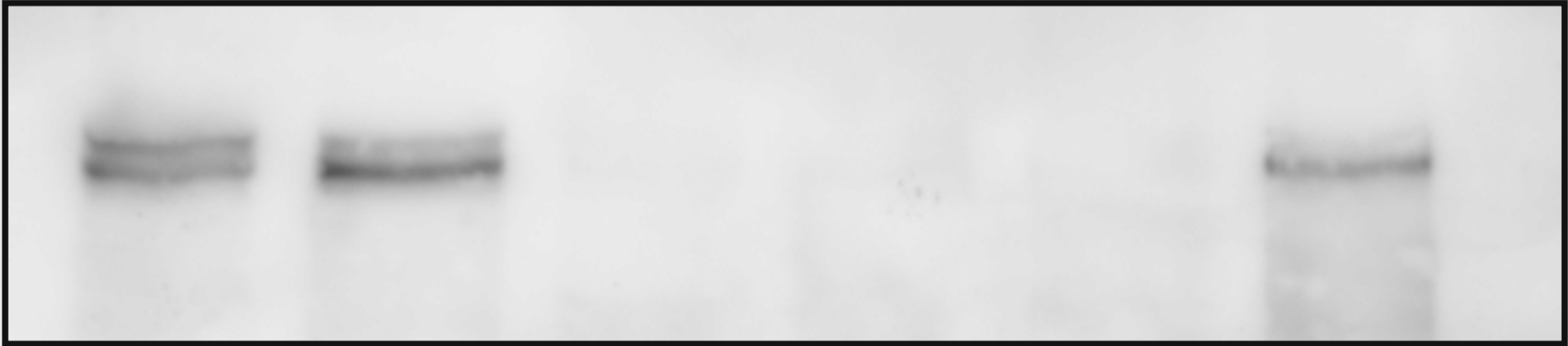

Supplement: Figure 5—figure supplement 1—source data 10. [file elife-88872-fig5-figsupp1-data10.zip › Figure 5-Source data 10/Slit2 S5c annotated.pdf]

myc antibody

| total input |   | IgG | IP: tomato |   | IgG | IP: tomato |               |
|-------------|---|-----|------------|---|-----|------------|---------------|
| +           | - |     | +          | - |     | +          | -             |
| +           | - | +   | +          | - | +   | +          | Tomato        |
| -           | + | -   | -          | + | -   | +          | N-Cad::Tomato |
| +           | + | +   | +          | + | +   | +          | Slit-2::Myc   |

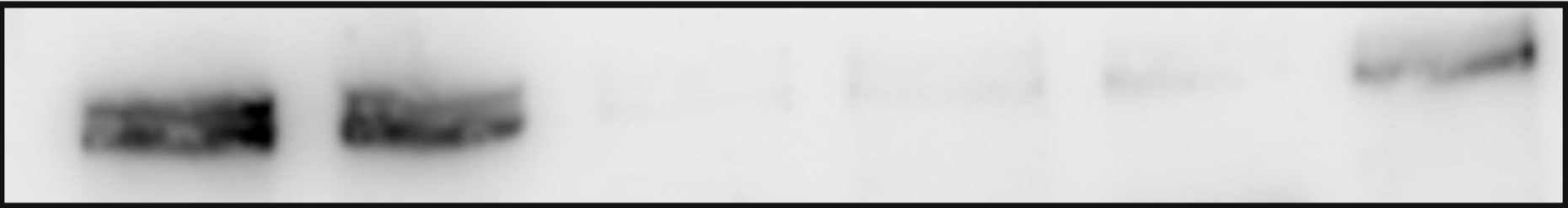

Supplement: Figure 5—figure supplement 1—source data 12. [file elife-88872-fig5-figsupp1-data12.zip › Figure 5-Source data 12/myc Figure 5c annotated.pdf]

tomato antibody

| <u>total input</u> |   | IgG | IP:    |   | IgG | IP:    |               |
|--------------------|---|-----|--------|---|-----|--------|---------------|
| +                  | - |     | tomato |   |     | tomato |               |
| +                  | - | +   | +      | - | -   | -      | Tomato        |
| -                  | + | -   | -      | + | +   | +      | N-Cad::Tomato |
| +                  | + | +   | +      | + | +   | +      | Slit-2::Myc   |

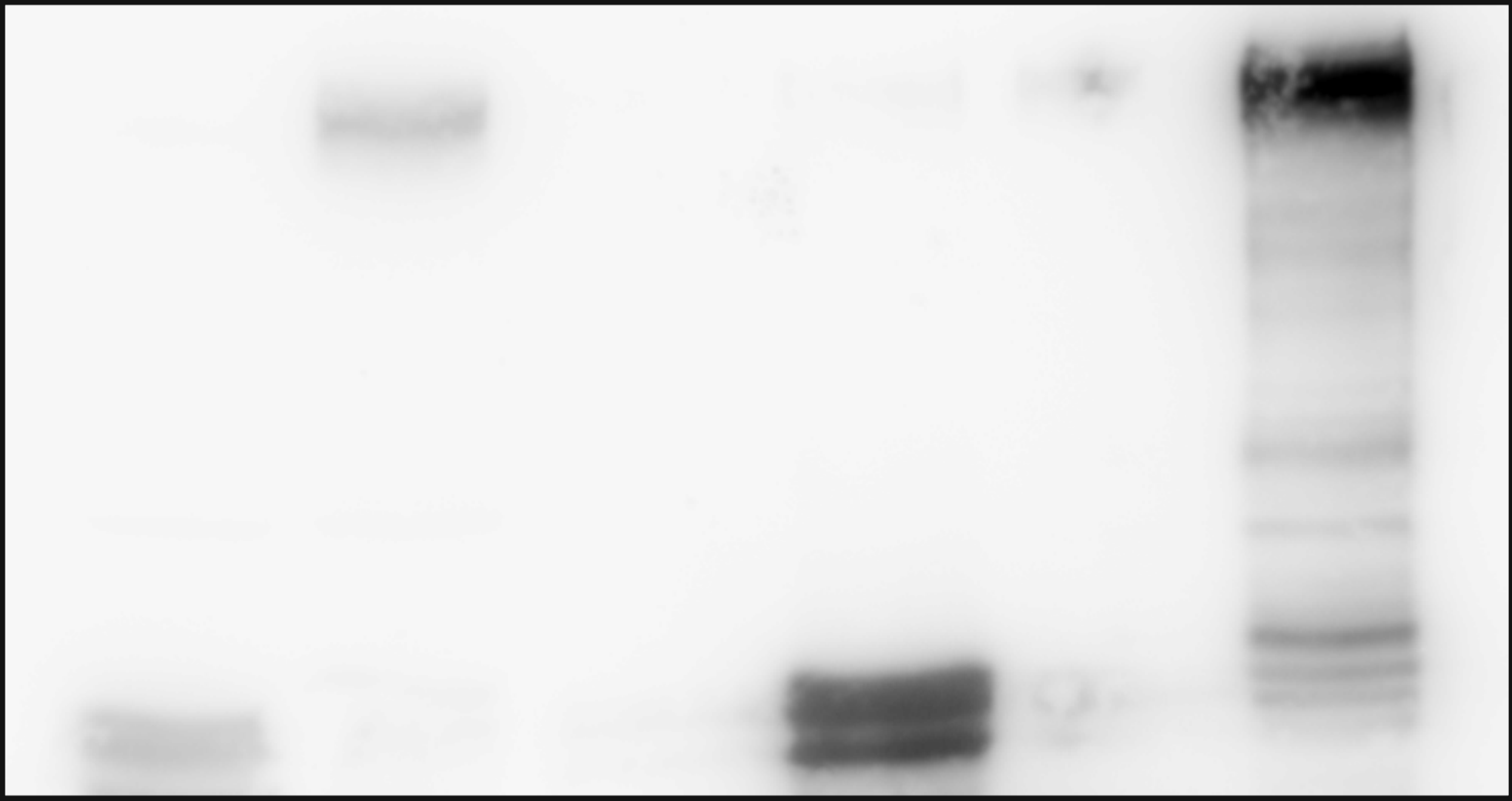

Supplement: Figure 5—figure supplement 1—source data 14. [file elife-88872-fig5-figsupp1-data14.zip › Figure 5-Source data 14/tomato S5c annotated.pdf]

Gel flipped in paper

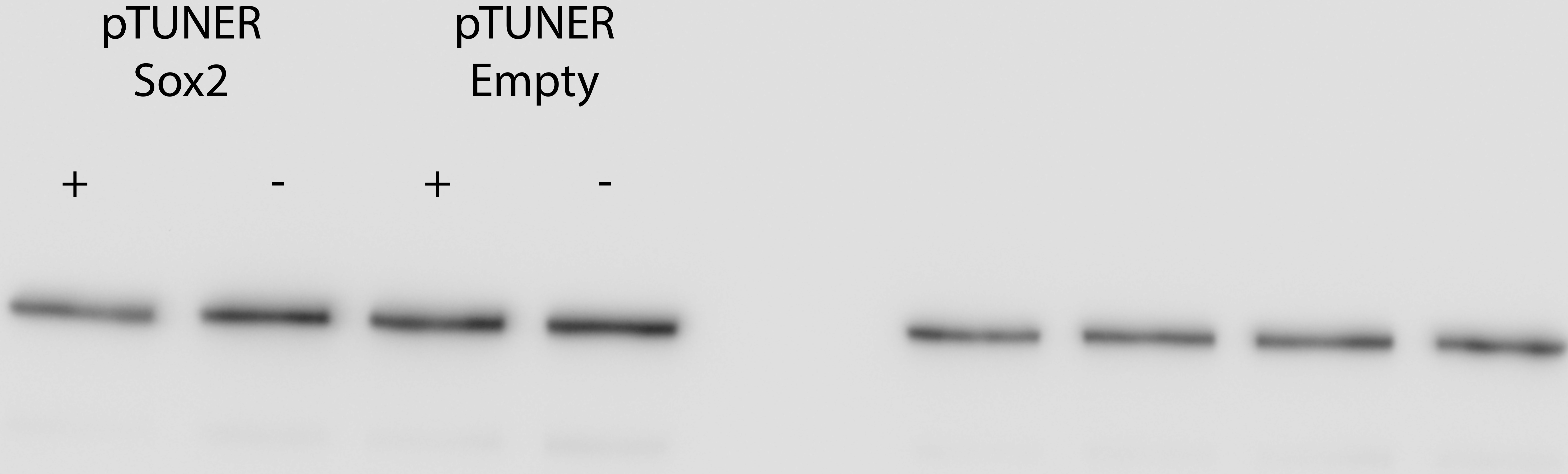

Supplement: Figure 6—figure supplement 1—source data 2. [file elife-88872-fig6-figsupp1-data2.zip › Figure 6-Source Data 2/N-cadherin S6c annotated.pdf]

Gel flipped in paper

pTUNER  
Sox2

+

-

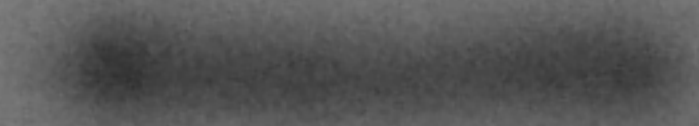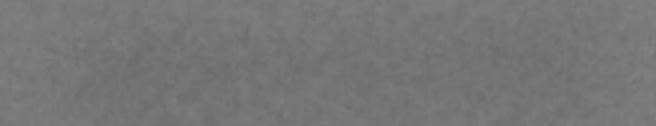

pTUNER  
Empty

+

-

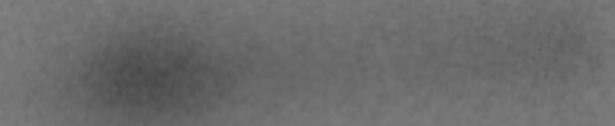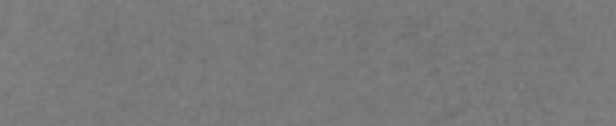

Supplement: Figure 6—figure supplement 1—source data 4. [file elife-88872-fig6-figsupp1-data4.zip › Figure 6-Source Data 4/S6c Sox2 annotated.pdf]

Gel flipped in paper

pTUNER  
Sox2

pTUNER  
Empty

+ - + -

Vinculin

alpha tubulin

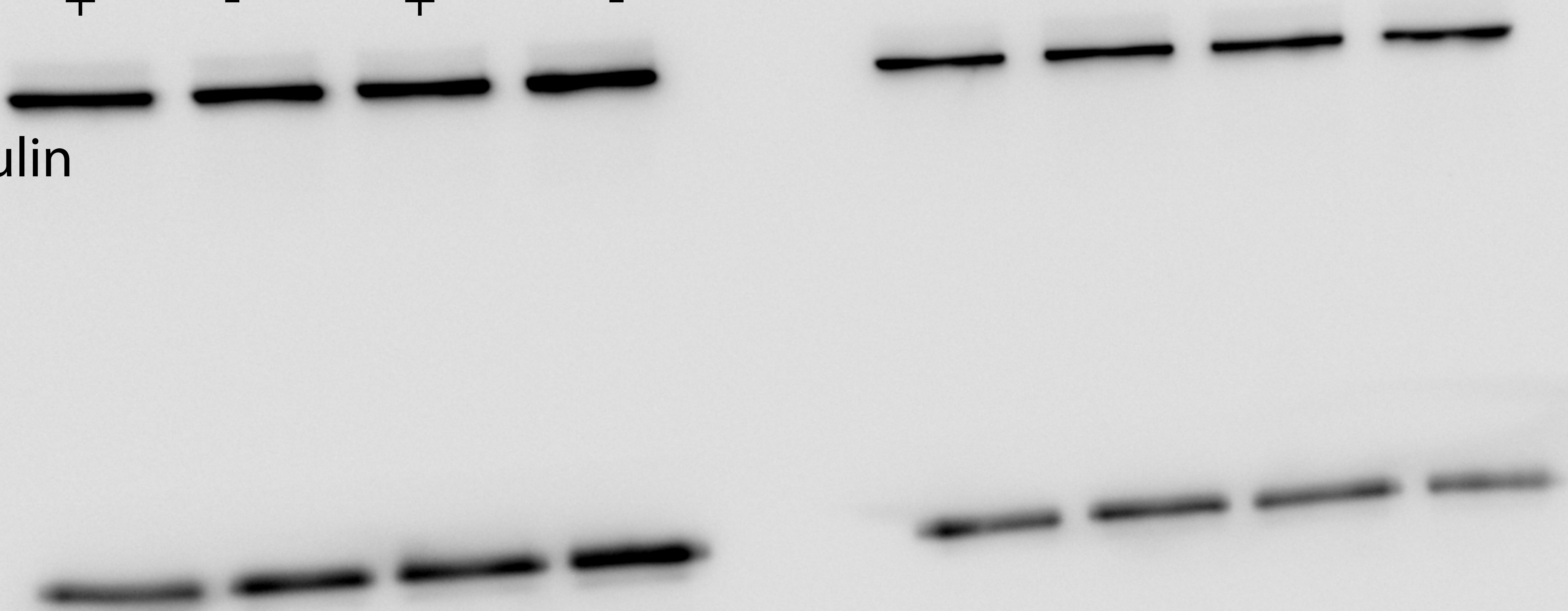

Supplement: Figure 6—figure supplement 1—source data 6. [file elife-88872-fig6-figsupp1-data6.zip › Figure 6-Source Data 6/Vinculin tubulin S6c annotated.pdf]
