# Supplementary material for: N-cadherin directs the collective Schwann cell migration required for nerve regeneration through Slit2/3-mediated contact inhibition of locomotion
Source: eLife. 2024 Apr 9;13:e88872. doi: 10.7554/eLife.88872 (PMC11052573; doi:10.7554/eLife.88872)
Supplement: Figure 4—figure supplement 1—source data 3. [file elife-88872-fig4-figsupp1-data3.zip › Figure 4-Source data 3/Robo4 gel annotated.pdf]

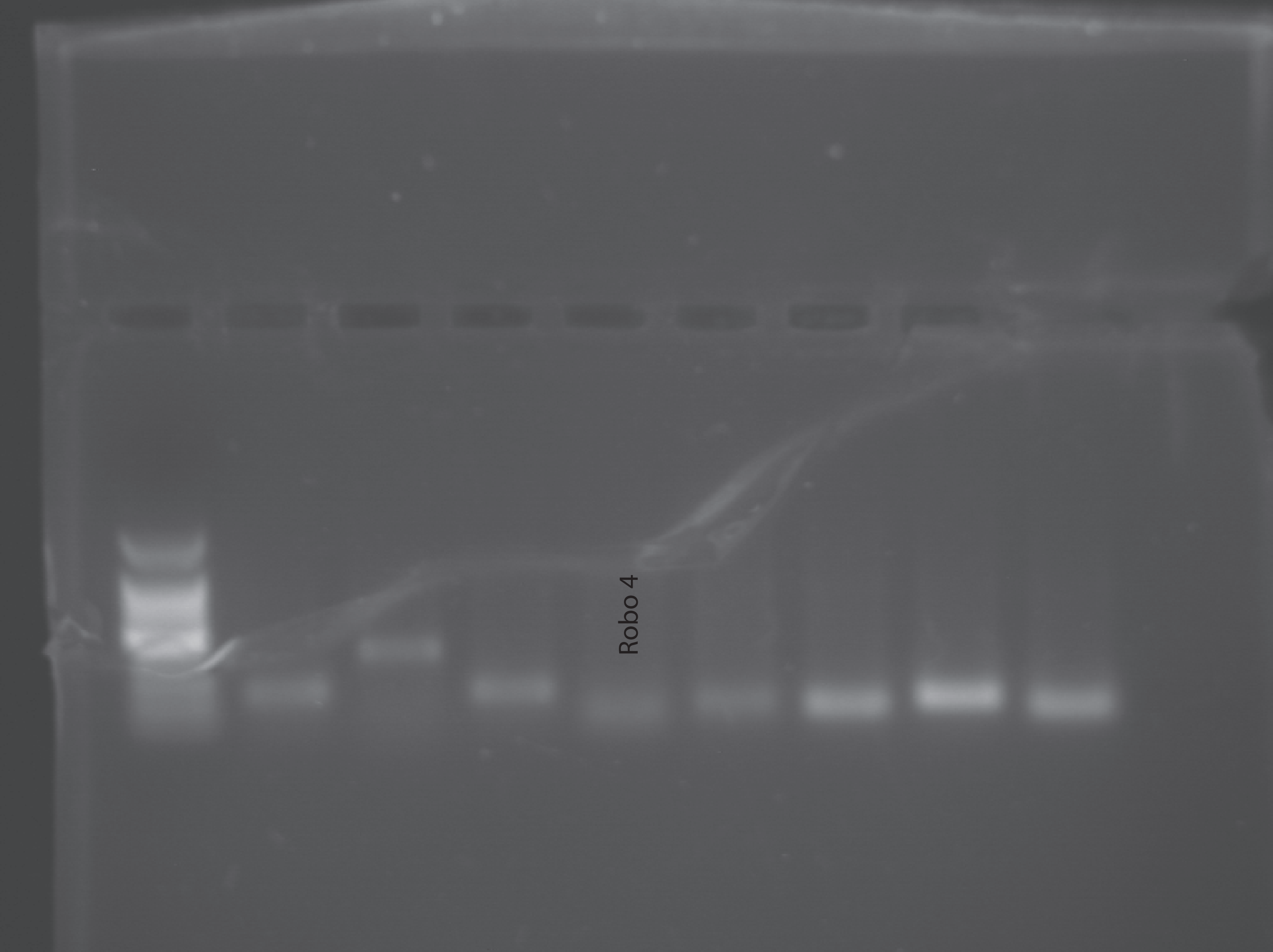
Robo 4
